# Influenza A(H5N1) Virus Infection in a Child With Encephalitis Complicated by Obstructive Hydrocephalus

**DOI:** 10.1093/cid/cix707

**Published:** 2017-08-07

**Authors:** Gannon Chun Kit Mak, Mike Yat-wah Kwan, Chris Ka Pun Mok, Janice Yee Chi Lo, Malik Peiris, Chi Wai Leung

**Affiliations:** 1 Public Health Laboratory Services Branch, Centre for Health Protection, Department of Health, University of Hong Kong; 2 HKU-Pasteur Research Pole, School of Public Health, University of Hong Kong; 3 Department of Paediatrics and Adolescent Medicine, Princess Margaret Hospital, Hong Kong Special Administrative Region, China

**Keywords:** avian influenza, central nervous system infection, H5N1, hydrocephalus

## Abstract

A 2-year-old boy with highly pathogenic avian influenza A(H5N1) virus infection with minimal respiratory symptoms developed encephalitis complicated by obstructive hydrocephalus. Viral RNA was detectable in cerebrospinal fluid. The virus belonged to H5N1 clade 2.3.2.1b and had acquired the mammalian adaptation mutation PB2 Q591K.

In the past 2 decades, highly pathogenic avian influenza (HPAI) A(H5N1) has become entrenched in poultry in a number of countries in Asia, the Middle East (eg, Egypt), and West Africa. Some clades of H5N1, related H5N8 viruses, and their descendants have been carried by wild-bird migration to Europe and North America. The zoonotic and pandemic risks associated with these viruses need to be continually monitored [1].

## CASE REPORT

A 2-year-old boy developed fever, runny nose, and productive cough on 23 May 2012 (day 1 of illness) in Guangdong province. He returned to Hong Kong on 26 May and sought outpatient medical attention. On 28 May (day 6 of illness), he developed convulsions and was admitted to the Caritas Medical Centre with a diagnosis of suspected encephalitis. His chest radiograph was unremarkable. A nasopharyngeal aspirate was found to be positive for influenza A by real-time reverse-transcription polymerase chain reaction (RT-PCR), negative for H1 and H3, and subsequently subtyped to be H5N1 [2]. The child was transferred to the Intensive Care Unit of the Infectious Disease Centre at Princess Margaret Hospital for isolation and further management. Oseltamivir therapy was commenced on 30 May (day 8 of illness) at a dose of 60 mg orally twice daily for a child weighing 11.9 kg—that is, double the dose recommended for uncomplicated influenza. The parents denied consent for a lumbar puncture at this stage. Epidemiological investigation revealed that the boy had visited a sidewalk wet market with live poultry in Guangzhou in mid-May. The boy’s parents remained asymptomatic and clinical specimens collected from them were negative for influenza A.

**Figure 1.**
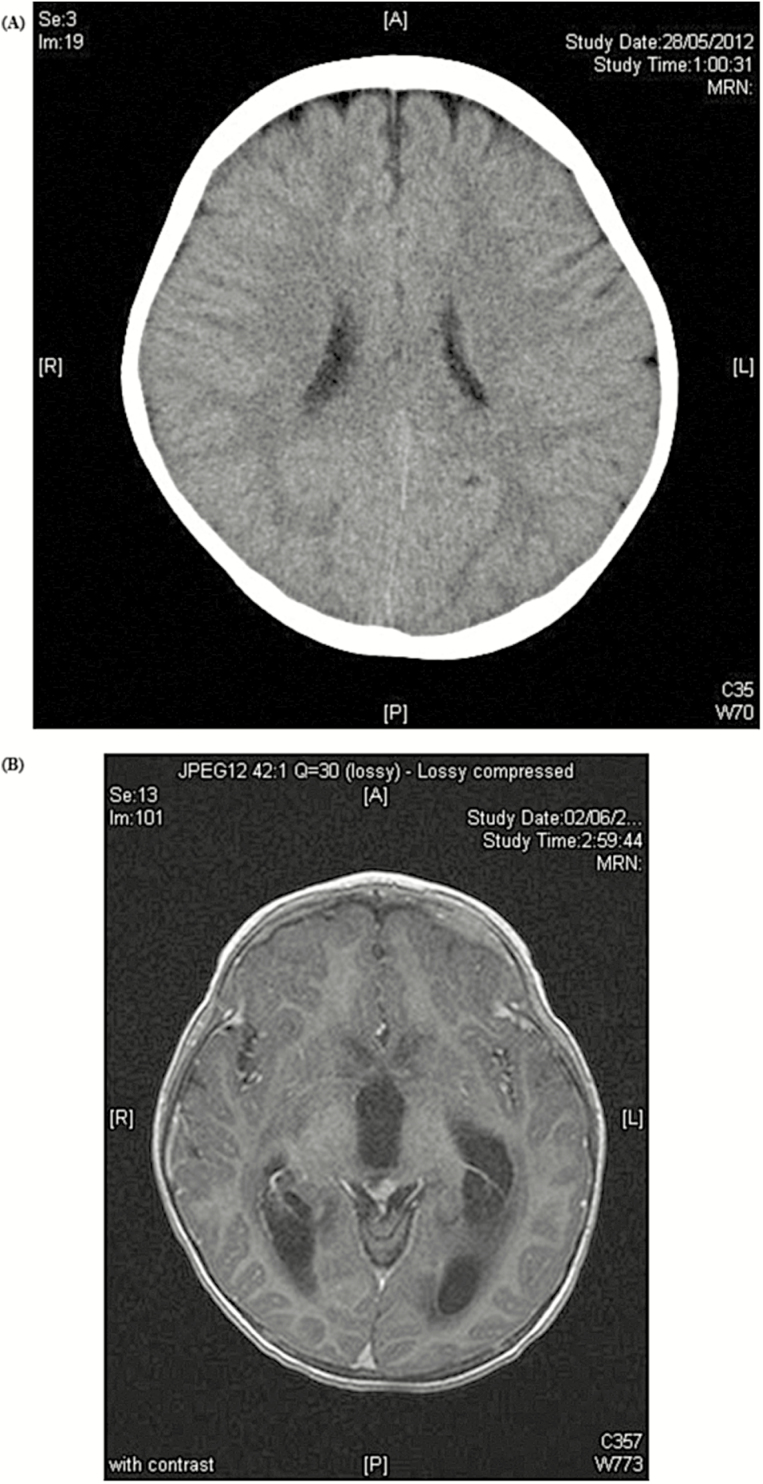
*A*, Computed tomography of the brain performed on day 6 of illness showed no focal lesion and no displacement of midline structures, and the ventricles were not dilated. *B*, Magnetic resonance imaging of the brain on day 11 of illness showed moderate obstructive hydrocephalus with involvement of the lateral and third ventricles.

Computed tomography of the brain performed on day 6 of illness showed no focal lesion and no displacement of midline structures, and the ventricles were not dilated (Figure 1A). Magnetic resonance imaging of the brain on day 11 of illness showed moderate obstructive hydrocephalus with transependymal flow of the cerebrospinal fluid (CSF) evidenced by the dilated bilateral lateral ventricles and the third ventricles (Figure 1B). The fourth ventricle was not dilated. The level of obstruction was at the cerebral aqueduct. An external ventricular catheter was inserted to relieve the obstructive hydrocephalus. Examination of the drained ventricular CSF on day 11 of illness revealed a red blood cell count 242 cells/μL, white blood cell count 165 cells/μL with lymphocyte predominance, protein 1.44 g/L, and glucose 2.4 mmol/L. A small fragment of brain tissue showed histological features of meningoencephalitis, and virus RNA was detectable at a borderline level (CT39). PCR for herpes simplex virus type 1 and 2 DNA and RT-PCR for enterovirus RNA were repeatedly negative in the CSF specimens and in the brain biopsy.

Serial specimens of CSF, nasopharyngeal aspirate (NPA), rectal swab, stool, urine, plasma, and serum were tested by real-time quantitative RT-PCR (qPCR) targeting M gene for influenza A virus and the hemagglutinin (HA) gene for H5N1 virus (Supplementary Methods; Table 1). The qPCR assay for the H5 gene was more sensitive than for the influenza M gene. CSF collected on day 11 and 13 as well as plasma on day 11 had detectable viral RNA (M gene and H5 gene), albeit at low viral load. In view of detection of virus RNA in CSF, the reported low CSF penetration of oseltamivir [3], known good intrathecal penetration of amantadine [4], and the virus genetic sequence (see below) without known mutations in the M2 gene associated with amantadine resistance, oral amantadine therapy (40 mg orally twice daily) was added to oseltamivir on day 14 of illness. The amantadine dosage was increased to 50 mg orally twice daily on day 27. In view of persistence of viral RNA in nasopharyngeal aspirates, ribavirin at a dose of 180 mg was given intravenously every 6 hours from day 27. The child received oseltamivir for 25 days, amantadine for 17 days, and ribavirin for 5 days. Given the late commencement of amantadine and ribavirin, their therapeutic contribution remains unclear. The ventricular drain was removed on day 35. The child made a complete recovery and was discharged from hospital on 5 July with satisfactory clinical condition. There were no neurological sequelae or developmental delay detected during subsequent follow-up.

**Table 1.** Serial Specimens Collected and Virological Results Obtained

Days Ill	RT-PCR Result (Cycle Threshold Value)												Clinical Comments
	NPA		Rectal Swab/ Feces		CSF		Brain Tissue		Urine		Serum/Plasma		
	M Gene	H5	M Gene	H5	M Gene	H5	M Gene	H5	M Gene	H5	M Gene	H5	
6	+ (25.6)^a^	+ (24.2)									+ (41.1)	–	
8											–	–	Commence oseltamivir
11	+ (30.9)^a^	+ (28.6)			+ (42.8)	+ (37.3)	-	+ (39.0)			+ (41.1)	+ (39.0)	Ventricular catheter inserted, CSF sampled
12											–	–	
13	+ (30.3)^a^	+ (28.2)			+ (40.9)	+ (37.9)			–	–			
14	+ (38.0)	+ (34.1)	–	–	–	–			–	–	–	–	Commence amantadine
15	+ (27.7)	+ (27.4)	–	–	–	–			–	–			
16	+ (33.1)	+ (31.3)	–	–	+ (41.6)	–			–	–			
17	+ (29.9)	+ (27.9)	–	–	–	–			–	–			
18	+ (39.7)	+ (36.4)											
19	+ (30.7)	+ (29.2)	–	–									
20	+ (35.9)	+ (34.3)			–	–							
21	+ (31.2)	+ (28.8)											
22	+ (30.0)	+ (28.2)			–	+ (40.0)	–	–					
23	+ (38.9)	+ (38.4)			–	–							
24	+ (38.1)	+ (37.1)	–	–	–	–							
25	+ (34.6)	+ (34.0)											
26	+ (36.8)	+ (34.1)											
27	–	–			–	–							Commence ribavirin
28	+ (37.9)	+ (34.9)											
29	+ (41.0)	+ (37.7)											
30	+ (38.9)	+ (37.7)											
31	+ (42.9)	+ (38.9)											
34					–	–							
35													Ventricular catheter removed

All specimens except for serum and plasma were also cultured for virus isolation. Cycle threshold value is inversely proportional to virus RNA load.

Abbreviations: +, positive; –, negative; CSF, cerebrospinal fluid; NPA, nasopharyngeal aspirate; RT-PCR, reverse-transcription polymerase chain reaction.

^a^Indicates virus isolate obtained.

Levels of monocyte chemoattractant protein 1, monokine induced by interferon gamma, and CXCL 10 (interferon gamma-induced protein 10) were assayed on the patient serum and CSF collected on day 11 of illness (Supplementary Methods). Serum samples from 6 controls (aged 6, 7, 8, 36, 42, and 47 years, collected from other noninfectious clinical indications and anonymized for chemokine testing) were also assayed (Supplementary Figure 1).

A madin darby canine kidney cell culture virus isolate designated A/Hong Kong/5923/2012 (H5N1) (abbreviated to H5N1/5923 hereafter) was obtained from an NPA specimen collected on day 6 of illness. The virus was fully genetically sequenced and phylogenetic analysis showed that the virus belonged to clade 2.3.2.1b in all 8 gene segments. The PB1 and PA gene segments of clade 2.3.2.1b viruses cluster also with corresponding genes of H5N6 viruses (Supplementary Figure 2). The H5N1/5923 virus had a Q591K amino acid substitution in the PB2 protein in specimens collected at day 6 and day 11 of illness. The HA1-HA2 connecting peptide possessed a multibasic amino acid sequence (PQIERRRRKR*GLF), which is common in clade 2.3.2.1b H5N1 viruses and characteristic of HPAI viruses. Mature HA protein has amino acid residues E190, Q226, and G228 (H3 numbering), indicating an avian-like receptor specificity (Supplementary Table) [5]. There were no mutations in the viral neuraminidase (NA) or M2 genes associated with resistance to NA inhibitors or adamantanes (Supplementary Table). The virus NA and M genes from NPA specimens collected on day 15, 17, 19, 21, and 22 of illness was also sequenced directly from the clinical specimen, and no known resistance mutations to NA inhibitors or adamantanes were detected (Supplementary Table).

## DISCUSSION

Patients infected with H5N1 present primarily with acute respiratory illness [6]. To our knowledge, there are only 2 other case reports of H5N1 infections with major manifestations of central nervous system (CNS) disease [7, 8] but neither with hydrocephalus. The first was a 4-year-old boy who presented with fever, headache, and diarrhea in Vietnam. He had no respiratory symptoms at initial admission and had a normal chest radiograph. He later developed a cough and had mild crackles and wheezing on auscultation. He became drowsy and then comatose. CSF examination revealed a moderately elevated CSF protein level but no increase in white cells. He progressed to respiratory failure and died. The diagnosis was acute encephalitis. Retrospective investigation as part of a study of encephalitis of unknown etiology yielded an influenza A(H5N1) virus isolate from the stored CSF specimen. The boy’s sister had died of a similar illness a few days previously [7]. A second patient was a young adult who presented with multilobar pneumonia progressing to diffuse encephalitis, leading to a fatal outcome [8].

HPAI H5N1 virus may enter the CNS via the blood circulation. Alternatively, virus from the nasopharynx may traverse the cribriform plate and enter the basal subarachnoid space with subsequent retrograde spread from the cisterna magna into the ventricular CSF space through the foramina of Luschka and Magendie. Spread of HPAI H5N1 virus to the brain along the olfactory nerves and via the cribriform plate has been reported in experimentally infected ferrets [9]. In this patient, inflammation resulting in acute ependymitis may have led to obliteration of the cerebral aqueduct between the third and fourth ventricles, the narrowest part of the ventricular system, resulting in obstructive hydrocephalus.

Patients infected with H5N1 reportedly rarely had detectable viral RNA in the respiratory tract for >3 weeks [6]. In the present study, viral RNA was detected in a patient with H5N1 from NPA up to day 30 after symptom onset. The RT-PCR cycle threshold value may be a useful indicator of response to therapy. The serum chemokine levels in the patient at day 11 after onset of illness were not markedly different to controls. Previous studies have reported that chemokine levels were higher in fatal rather than nonfatal patients with H5N1 disease [10, 11]. In this patient, pathology was primarily in the CNS rather than the lung; it is therefore interesting that the chemokine level in the patient’s CSF was markedly elevated in comparison to the level in the serum sample collected on the same day, providing additional evidence of inflammation within the CNS compartment (Supplementary Figure 1).

Previous studies have shown that specific amino acid substitutions in PB2 of avian influenza viruses enhance replication competence in mammalian cells and are recognized mammalian adaptation markers. In contrast to other clade 2.3.2.1 H5N1 viruses, the H5N1/5923 virus from our patient had a PB2 Q591K mutation. This amino acid substitution has been shown to increase the replication and pathogenicity of avian H5N1, H9N2, and H7N9 viruses in mammals in a manner analogous to previously reported mammalian adaptation signatures E627K and D701N [12, 13]. However, while the PB2-E627K mediates the virus dissemination to the brain after infection, a similar role for PB2-Q591K has not yet been reported in experimental mammalian models, although this remains plausible.

The genetic analysis of the virus HA suggests that the virus had a preference for binding α(2–3)–linked sialic acids found in avian species. Adult humans have a preponderance of α(2-6)–linked sialic acids in their upper respiratory tract, and H5N1 virus disease is believed to take place when the virus gains access to the alveolar epithelium where α(2-3)–linked sialic acids are present. However, it has been reported that young children differ in this respect and have a preponderance of α(2-3)–linked sialic acids in their upper respiratory tract [14]. These findings need independent confirmation. However, it is interesting to speculate whether this may have predisposed this child to infection with this avian virus infecting the upper respiratory tract and subsequently passing via the cribriform plate to invade the CNS. In this patient, it is possible that host (young age, genetic susceptibility) or viral determinants, or both, contributed to the unusual CNS manifestations.

In conclusion, we report influenza A(H5N1) infection in a child presenting as encephalitis and progressing to obstructive hydrocephalus, preceded by mild upper respiratory symptoms. In many instances, such patients may be primarily investigated for typical causes of CNS disease rather than for influenza A. This case report highlights the need to have a high index of suspicion and the need to investigate patients with CNS disease for HPAI H5N1 in areas where this virus is endemic in poultry and in travelers returning from such areas, even when respiratory symptoms are not the prominent presenting feature.

## Supplementary Data

Supplementary materials are available at *Clinical Infectious Diseases* online. Consisting of data provided by the authors to benefit the reader, the posted materials are not copyedited and are the sole responsibility of the authors, so questions or comments should be addressed to the corresponding author.

## Supplementary Material

Supplementary Methods online

Supplementary Figure 1

Supplementary Figure 2

Supplementary Table

## Notes

***Financial support.*** This work was supported by research grants from the US National Institute of Allergy and Infectious Diseases (NIAID) under Centers of Excellence for Influenza Research and Surveillance contract number HHSN272201400006C; and by the Research Grants Council of the Hong Kong Special Administrative Region, China, through the Theme-Based Research Scheme (grant number T11-705/14N to M. P.).

***Potential conflicts of interest.*** All authors: No reported conflicts of interest. All authors have submitted the ICMJE form for disclosure of potential conflicts of interest. Conflicts that the editors consider relevant to the content of the manuscript have been disclosed.
