# Supplementary material for: Influenza A(H5N1) Virus Infection in a Child With Encephalitis Complicated by Obstructive Hydrocephalus
Source: Clin Infect Dis. 2017 Aug 7;66(1):136–9. doi: 10.1093/cid/cix707 (PMC5850530; doi:10.1093/cid/cix707)
Supplement: Supplementary Methods online [file cix707_suppl_supplementary_methods_online.doc]

**Supplementary data online:**

Supplementary methods online: Data on RT-PCR diagnostic methods, cytokine assays and phylogenetic analysis methods

Supplementary figure 1 online: Levels of pro-inflammatory chemokines MCP-1, MIG and CSCL-10 (IP-10) in the serum and CSF of the patient on day 11 of illness and in serum of 6 health controls.

Supplementary figure 2 online: Phylogenetic analysis of the eight gene segments of the virus isolate. Neighbor-Joining trees were constructed for each gene segment using MEGA software. Similar sequences except clade 2.3.2.1a-c are collapsed in triangle. GISAID accession numbers for each gene segment are: PB2: EPI375501; PB1: EPI375502; PA: EPI375503; HA: EPI375432; NP: EPI375504; NA: EPI375433; M: EPI375434; NS: EPI375506.

Supplementary table online: Key molecular signatures of A/Hong Kong/5923/2012 (H5N1) known to contribute to pathogenicity or antiviral resistance.

**Supplementary information online methods:**

Real-time quantitative RT-PCRs: Detection of influenza A(H5N1) virus in clinical specimens was carried out by RT-PCR targeting either M gene of influenza A viruses or HA gene of influenza A(H5N1) viruses. The cDNA synthesis, primers and probes used and thermal cycling conditions was based on WHO approved methods reported previously [1].

Quantitative analysis of cytokines: Protein levels of CXCL10 (IP-10), MIG and MCP-1 from the serum were quantitatively determined by flow cytometry-based immunoassay using a CBA human inflammatory cytokine kit (BD Biosciences, San Jose, CA, USA) according to the manufacturer’s instructions. Data acquisition was performed on BD LSR Fortessa (BD Biosciences). Data were analyzed by CBA analysis software.

Phylogenetic analysis: To assess the phylogenetic relationship of A/Hong Kong/5923/2012 (H5N1) to other circulating H5N1 viruses and related novel H5N6 viruses detected in China, all 8 gene segments of this virus were genetically sequenced (GISAID accession numbers: PB2: EPI375501; PB1: EPI375502; PA: EPI375503; HA: EPI375432; NP: EPI375504; NA: EPI375433; M: EPI375434; NS: EPI375506). Only viruses with full-length open reading frame in all 8 gene segments were included in the phylogenetic analysis. All available H5N6 sequences except the NA gene were also aligned together with H5N1 sequences. Representative H5N1 and H5N6 virus sequences were included in the phylogentic analysis. Only viruses with full-length open reading frame in all 8 gene segments were included in the phylogenetic analysis. All available H5N6 sequences except the NA gene were also aligned together with H5N1 sequences. Sequence editing, alignment and alignment trimming for each gene segment were performed with the BioEdit software. Each gene segment except the HA gene was trimmed to the coding region from start codon to end codon. The HA gene was trimmed to the beginning of the mature H5 HA protein gene sequence according to the previous study [2]. A total of 154 H5N1 and 131 H5N6 full genomes were downloaded from GenBank and GISAID. Neighbor-Joining trees were constructed for each gene segment using MEGA software. Similar sequences except clade 2.3.2.1a-c are collapsed in triangle. Clade affiliations of all H5N1 viruses assigned in the HA phylogenetic tree were used in the phylogenetic trees of the other seven genes. Each leaf node contains two sections: designated name of isolate and clade affiliations assigned in the HA phylogenetic tree in parenthesis.

^1^ **WHO.** Recommendations and laboratory procedures for detection of avian influenza A(H5N1) virus in specimens from suspected human cases, Revised August 2007; page 15-16. Available at: <http://www.who.int/influenza/resources/documents/h5n1_laboratory_procedures/en/> (Accessed March 7th 2017)

^2^ **World Health Organization/World Organization for Animal Health/Food and Agriculture Organization (WHO/OIE/FAO) H5N1 Evolution Working Group. 2013.** Revised and updated nomenclature for highly pathogenic avian influenza A (H5N1) viruses. *Influenza Other Respir Viruses* **8** 384-838.
