# Supplementary figures and images for: Influenza A(H5N1) Virus Infection in a Child With Encephalitis Complicated by Obstructive Hydrocephalus
Source: Clin Infect Dis. 2017 Aug 7;66(1):136–9. doi: 10.1093/cid/cix707 (PMC5850530; doi:10.1093/cid/cix707)

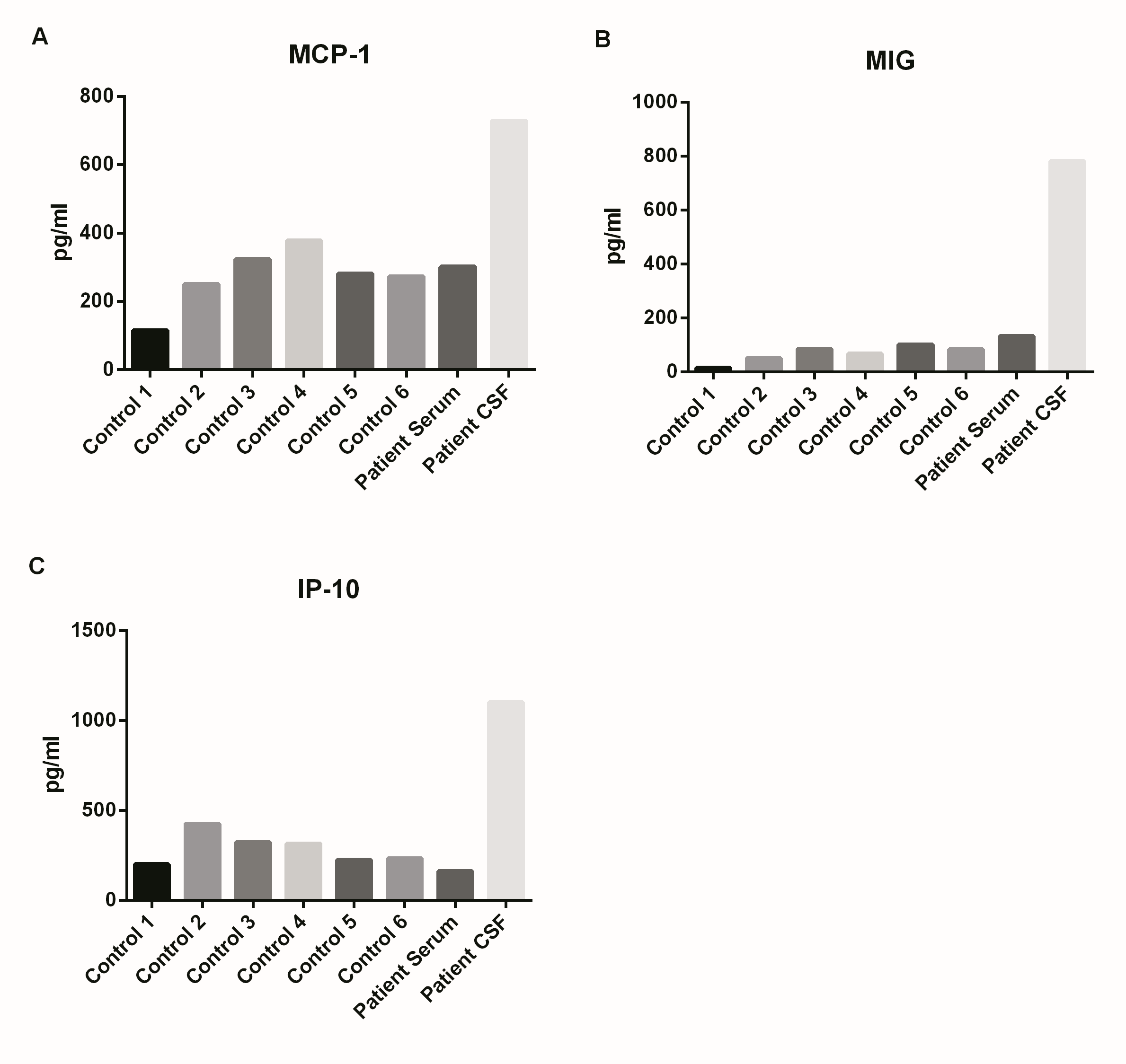

Supplement: Supplementary Figure 1 [file cix707_suppl_supplementary_figure_1.png]
