# Supplementary material for: Influenza A(H5N1) Virus Infection in a Child With Encephalitis Complicated by Obstructive Hydrocephalus
Source: Clin Infect Dis. 2017 Aug 7;66(1):136–9. doi: 10.1093/cid/cix707 (PMC5850530; doi:10.1093/cid/cix707)

Phylogeny of PB2 gene

- H5N1 viruses: no dots
- H5N1 viruses clade 2.3.2.1a: red dots
- H5N1 viruses clade 2.3.2.1b: green dots
- H5N1 viruses clade 2.3.2.1c: yellow dots
- H5N6 viruses: grey dots
- the 5923 H5N1 virus from human: blue dot

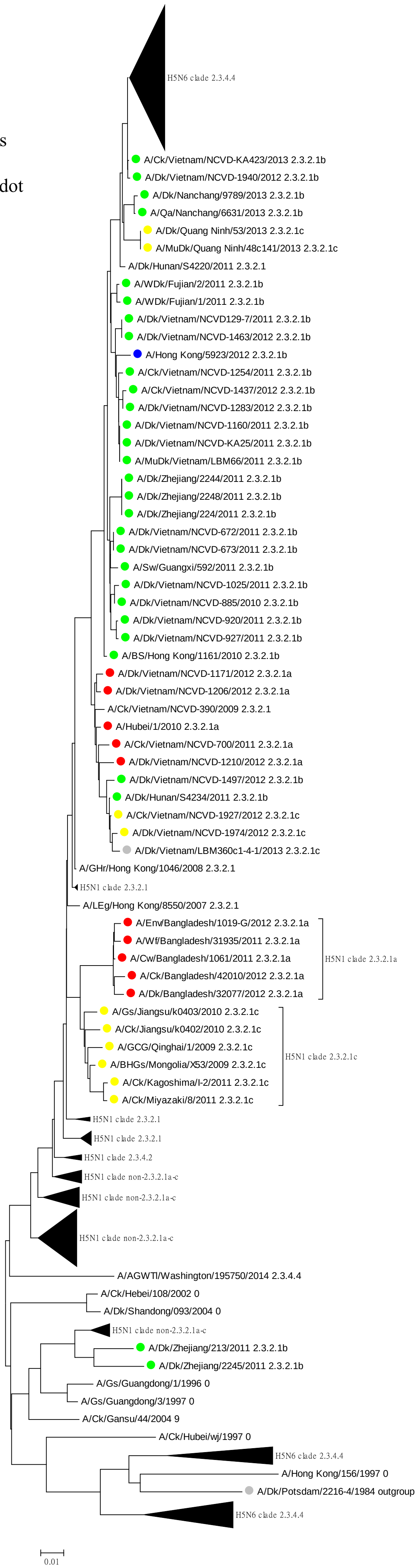

Phylogeny of PB1 gene

- H5N1 viruses: no dots
- H5N1 viruses clade 2.3.2.1a: red dots
- H5N1 viruses clade 2.3.2.1b: green dots
- H5N1 viruses clade 2.3.2.1c: yellow dots
- H5N6 viruses: grey dots
- the 5923 H5N1 virus from human: blue dot

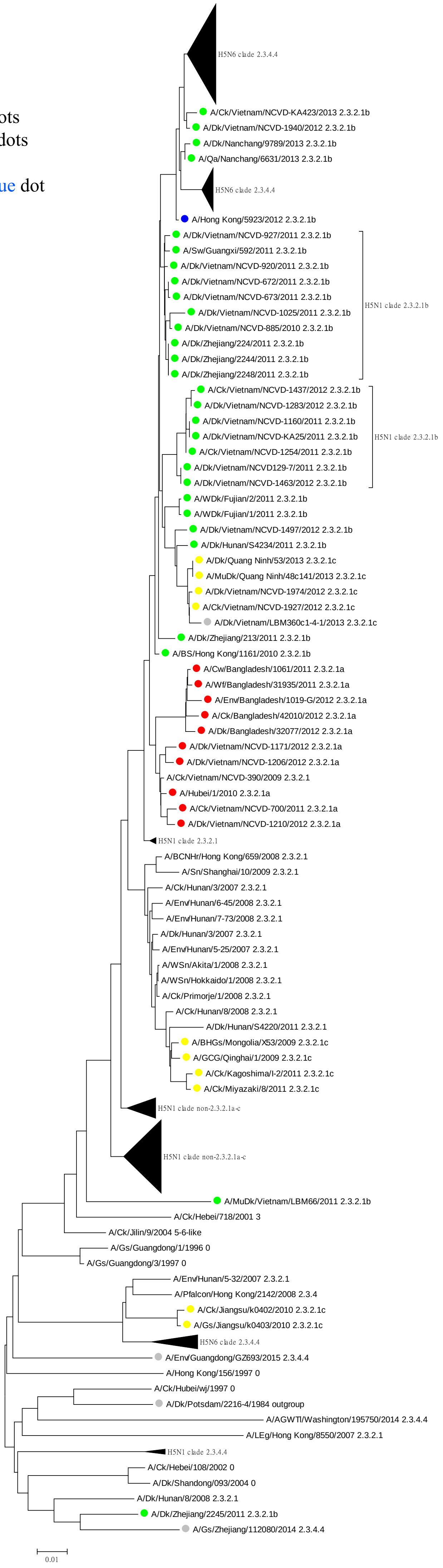

Phylogeny of PA gene

- H5N1 viruses: no dots
- H5N1 viruses clade 2.3.2.1a: red dots
- H5N1 viruses clade 2.3.2.1b: green dots
- H5N1 viruses clade 2.3.2.1c: yellow dots
- H5N6 viruses: grey dots
- the 5923 H5N1 virus from human: blue dot

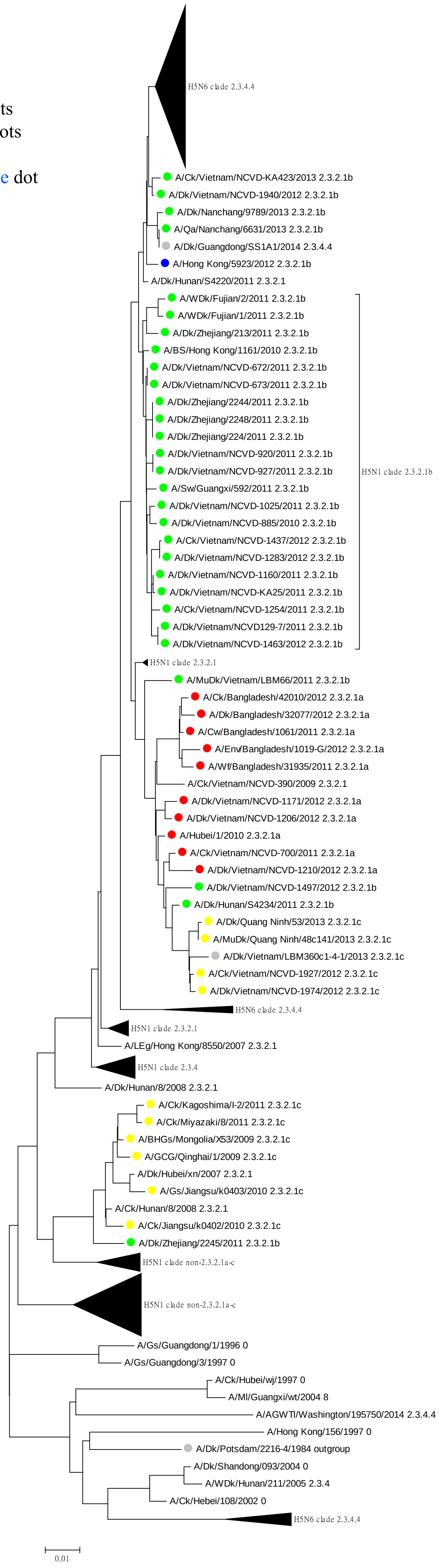

Phylogeny of H5 gene

- H5N1 viruses: no dots
- H5N1 viruses clade 2.3.2.1a: red dots
- H5N1 viruses clade 2.3.2.1b: green dots
- H5N1 viruses clade 2.3.2.1c: yellow dots
- H5N6 viruses: grey dots
- the 5923 H5N1 virus from human: blue dot

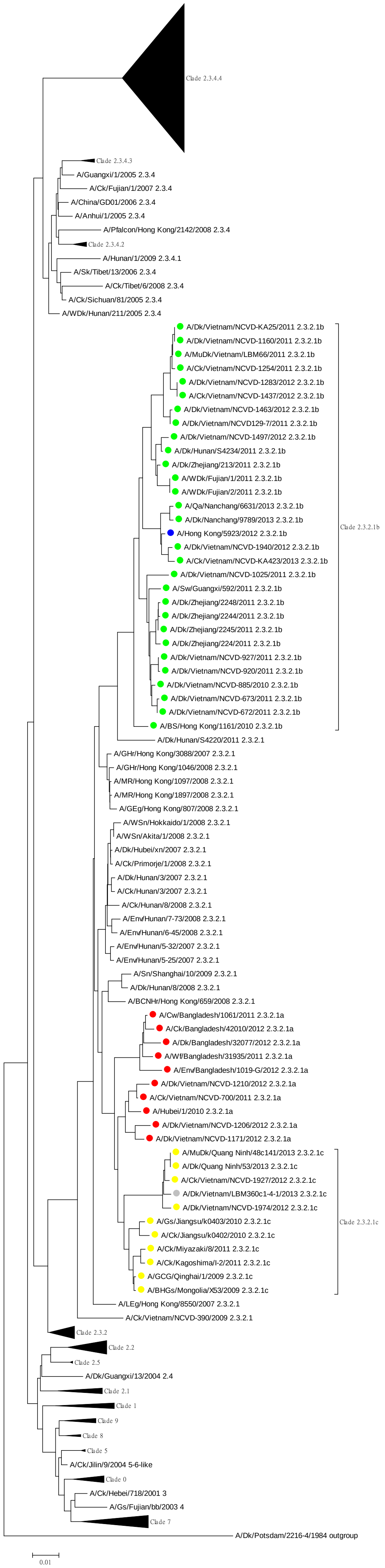

Phylogeny of NP gene

- H5N1 viruses: no dots
- H5N1 viruses clade 2.3.2.1a: red dots
- H5N1 viruses clade 2.3.2.1b: green dots
- H5N1 viruses clade 2.3.2.1c: yellow dots
- H5N6 viruses: grey dots
- the 5923 H5N1 virus from human: blue dot

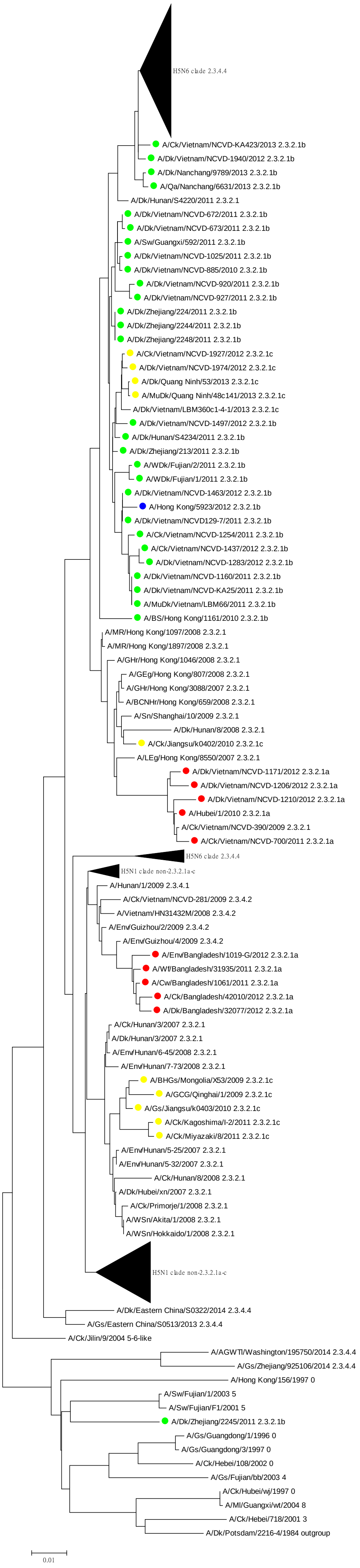

Phylogeny of N1 gene

- H5N1 viruses: no dots
- H5N1 viruses clade 2.3.2.1a: red dots
- H5N1 viruses clade 2.3.2.1b: green dots
- H5N1 viruses clade 2.3.2.1c: yellow dots
- the 5923 H5N1 virus from human: blue dot

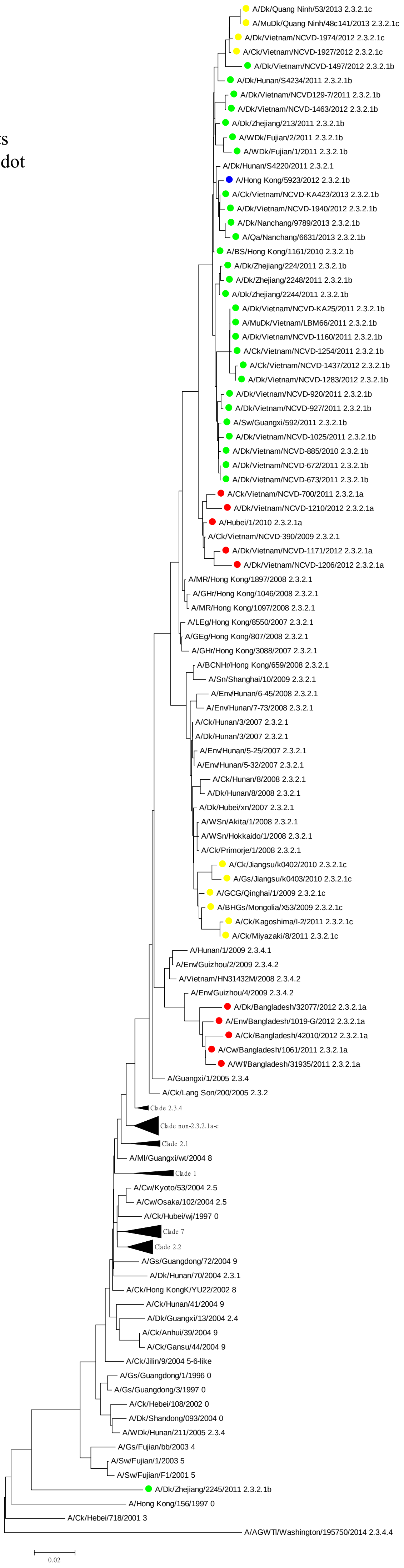

Phylogeny of M gene

- H5N1 viruses: no dots
- H5N1 viruses clade 2.3.2.1a: red dots
- H5N1 viruses clade 2.3.2.1b: green dots
- H5N1 viruses clade 2.3.2.1c: yellow dots
- H5N6 viruses: grey dots
- the 5923 H5N1 virus from human: blue dot

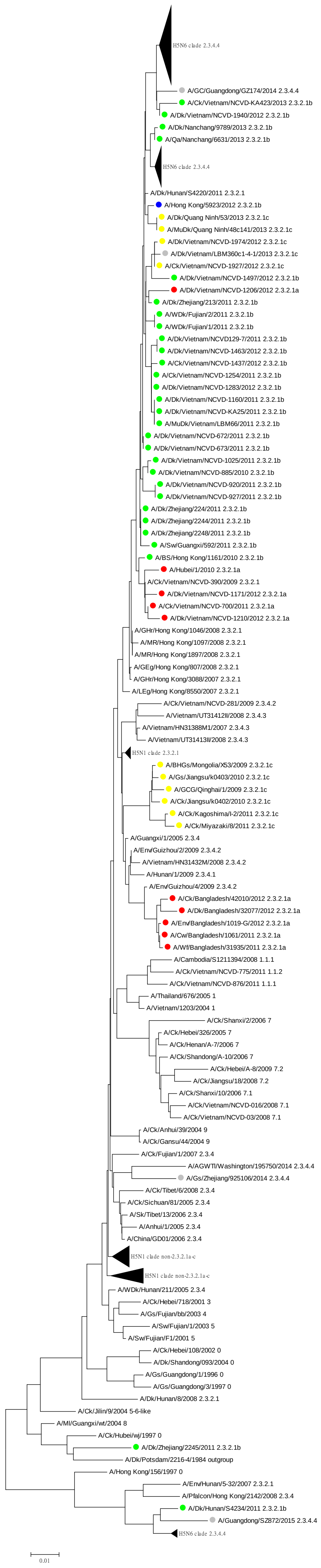

Phylogeny of NS gene

- H5N1 viruses: no dots
- H5N1 viruses clade 2.3.2.1a: red dots
- H5N1 viruses clade 2.3.2.1b: green dots
- H5N1 viruses clade 2.3.2.1c: yellow dots
- H5N6 viruses: grey dots
- the 5923 H5N1 virus from human: blue dot

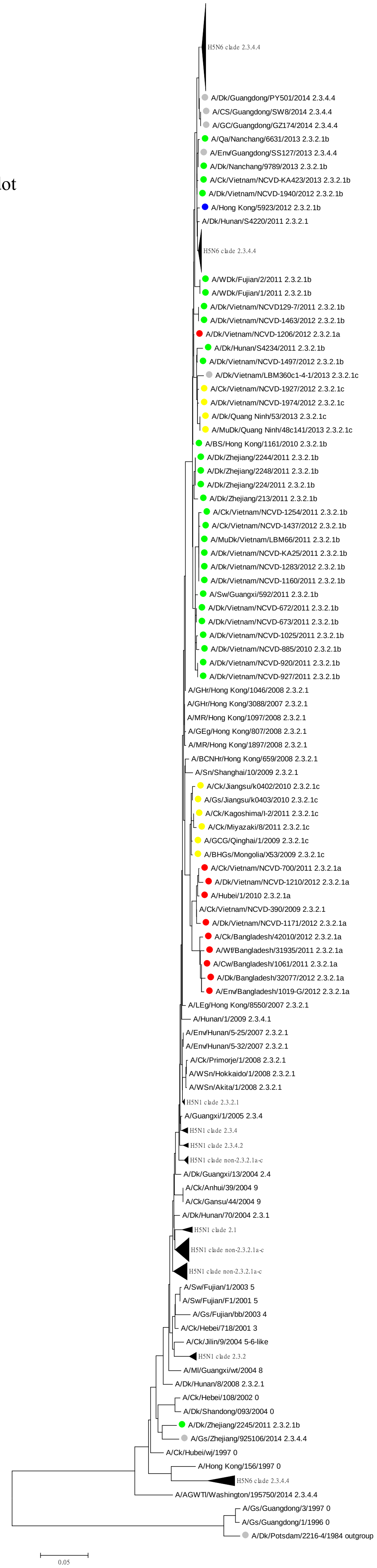

Supplement: Supplementary Figure 2 [file cix707_suppl_supplementary_figure_2.pdf]
