# Supplementary material for: Influenza A(H5N1) Virus Infection in a Child With Encephalitis Complicated by Obstructive Hydrocephalus
Source: Clin Infect Dis. 2017 Aug 7;66(1):136–9. doi: 10.1093/cid/cix707 (PMC5850530; doi:10.1093/cid/cix707)
Supplement: Supplementary Table [file cix707_suppl_supplementary_table.docx]

**Supplementary table: Key molecular signatures of A/Hong Kong/5923/2012 (H5N1)**

| Influenza segment^1^ | H5N1/5923 | Reference^4^ |
| --- | --- | --- |
| **1. Enhanced replication efficiency / virulence** |  |  |
| *PB2* |  |  |
| - Q591K | K | Yamada et al., 2010 |
| - E627K | E | Hatta et al., 2001; Mase et al., 2006; Hatta et al., 2007; Chen et al., 2007; Fornek et al., 2009 |
| - D701N | D | Li et al., 2005 |
| *PB1-F2* |  |  |
| - 87-90 amino acids in length | 57 amino acids in length | Smith et al., 2013 |
| *NA* |  |  |
| - Amino acids 49-68 deletion^2^ | Amino acids 49-68 deletion^3^ | Matsuoka et al., 2009; Zhou et al., 2009 |
| *NS1* |  |  |
| - P42S | S^3^ | Jiao et al., 2008 |
| - Amino acids 80-84 deletion^2^ | Amino acids 80-84 deletion^3^ | Long et al., 2008 |
| - D87E | E^3^ | Seo et al., 2002 |
| - L98F | F^3^ | Kuo et al., 2009; Spesock et al., 2011 |
| - I101M | M^3^ | Kuo et al., 2009; Spesock et al., 2011 |
| - PDZ domain (X-S/T-X-V at positions 222-225) | ESEV^3^ | Jackson et al., 2008 |
| **2. Enhanced binding to α2,6** |  |  |
| *HA*, H5 numbering |  |  |
| - N182K (186 in H3) | N | Yamada et al., 2006; Chutinimitkul et al., 2010 |
| - V210I (214 in H3) | V | Watanabe et al., 2011 |
| - Q222L (226 in H3) | Q | Chutinimitkul et al., 2010 |
| - S223N (227 in H3) | R | Chen et al., 2012 |
| - G224S (228 in H3) | G | Chutinimitkul et al., 2010 |
| - N154D, N220K, Q222L, T315I (158, 224, 226, 318 in H3) | D, N, Q, T | Imai et al., 2012 |
| **3. Reduced susceptibility to neuraminidase inhibitors** |  |  |
| *NA*, N1 numbering |  |  |
| - V96A (116 in N2) | V | Hurt et al., 2007; Boltz et al., 2010 |
| - I97V (117 in N2) | I | Hurt et al., 2007; Ilyushina et al., 2010; Le et al., 2008 |
| - E99A, E99G (119 in N2) | E | Hurt et al., 2009; Ilyushina et al., 2010 |
| - Q116L (136 in N2) | Q | Hurt et al., 2010 |
| - V129A (149 in N2) | V | Naughtin et al., 2011 |
| - R136K (156 in N2) | R | Ilyushina et al., 2012 |
| - D179G (198 in N2) | D | Hurt et al., 2009 |
| - I203M (222 in N2) | I | Hurt et al., 2009 |
| - S227N (246 in N2) | S | Boltz et al., 2010 |
| - H255Y (274 in N2) | H | Le et al., 2005; de Jong et al., 2005 |
| - N275S (294 in N2) | N | Le et al., 2005; Yen et al., 2007; Earhart et al.,2009; Kiso et al., 2011 |
| - I97V+I294V (117+314 in N2) | I, I | WHO |
| - K130N+I203L+S227N (150+222+246 in N2) | K, I, S | WHO |
| **4. Reduced susceptibility to adamantanes** |  |  |
| *M2* |  |  |
| - V27A | V | Cheung et al., 2006; Ilyushina et al., 2005 |
| - A30S | A | Cheung et al., 2006; |
| - S31N | S | Cheung et al., 2006; He et al., 2008; Ilyushina et al., 2005; Puthavathana et al.,2005; Buranathai et al., 2007 |

^1^ The amino acid positions are relative to A/Vietnam/1203/2004 (H5N1) unless specified.

^2^ Relative to A/goose/Guangdong/1/1996 (H5N1).

^3^ Conserved among contemporary H5N1 viruses.

^4^ Laboratory studies related to H5N1 viruses.

**Boltz DA, Douangngeun B, Phommachanh P, Sinthasak S, Mondry R, Obert C, Seiler P, Keating R, Suzuki Y, Hiramatsu H, Govorkova EA, Webster RG. 2010.** Emergence of H5N1 avian influenza viruses with reduced sensitivity to neuraminidase inhibitors and novel reassortants in Lao People's Democratic Republic. *J Gen Virol* **91** 949-959.

**Buranathai C, Amonsin A, Chaisigh A, Theamboonlers A, Pariyothorn N, Poovorawan Y. 2007.** Surveillance activities and molecular analysis of H5N1 highly pathogenic avian influenza viruses from Thailand, 2004-2005. *Avian Dis* **51** 194-200.

**Chen H, Bright RA, Subbarao K, Smith C, Cox NJ, Katz JM, Matsuoka Y. 2007.** Polygenic virulence factors involved in pathogenesis of 1997 Hong Kong H5N1 influenza viruses in mice. *Virus Res* **128** 159-163.

**Chen LM, Blixt O, Stevens J, Lipatov AS, Davis CT, Collins BE, Cox NJ, Paulson JC, Donis RO. 2012.** In vitro evolution of H5N1 avian influenza virus toward human-type

receptor specificity. *Virology* **422** 105-113.

**Cheung CL, Rayner JM, Smith GJ, Wang P, Naipospos TS, Zhang J, Yuen KY, Webster RG, Peiris JS, Guan Y, Chen H. 2006.** Distribution of amantadine-resistant H5N1 avian influenza variants in Asia. *J Infect Dis* **193** 1626-1629.

**Chutinimitkul S, van Riel D, Munster VJ, van den Brand JM, Rimmelzwaan GF, Kuiken T, Osterhaus AD, Fouchier RA, de Wit E. 2010.** In vitro assessment of attachment pattern and replication efficiency of H5N1 influenza A viruses with altered receptor specificity. *J Virol* **84** 6825-6833.

**de Jong MD, Tran TT, Truong HK, Vo MH, Smith GJ, Nguyen VC, Bach VC, Phan TQ, Do QH, Guan Y, Peiris JS, Tran TH, Farrar J. 2005.** Oseltamivir resistance during treatment of influenza A (H5N1) infection. *N Engl J Med* **353** 2667-2672.

**Earhart KC, Elsayed NM, Saad MD, Gubareva LV, Nayel A, Deyde VM, Abdelsattar A, Abdelghani AS, Boynton BR, Mansour MM, Essmat HM, Klimov A, Shuck-Lee D, Monteville MR, Tjaden JA. 2009.** Oseltamivir resistance mutation N294S in human influenza A(H5N1) virus in Egypt. *J Infect Public Health* **2** 74-80.

**Fornek JL, Gillim-Ross L, Santos C, Carter V, Ward JM, Cheng LI, Proll S, Katze MG, Subbarao K. 2009.** A single-amino-acid substitution in a polymerase protein of an H5N1 influenza virus is associated with systemic infection and impaired T-cell activation in mice. *J Virol* **83** 11102-11115.

**Hatta M, Gao P, Halfmann P, Kawaoka Y. 2001.** Molecular basis for high virulence of Hong Kong H5N1 influenza A viruses. *Science* **293** 1840-1842.

**Hatta M, Hatta Y, Kim JH, Watanabe S, Shinya K, Nguyen T, Lien PS, Le QM, Kawaoka Y. 2007.** Growth of H5N1 influenza A viruses in the upper respiratory tracts of mice. *PLoS Pathog* **3** 1374-1379.

**He G, Qiao J, Dong C, He C, Zhao L, Tian Y. 2008.** Amantadine-resistance among H5N1 avian influenza viruses isolated in Northern China. *Antiviral Res* **77** 72-76.

**Hurt AC, Selleck P, Komadina N, Shaw R, Brown L, Barr IG. 2007.** Susceptibility of highly pathogenic A(H5N1) avian influenza viruses to the neuraminidase inhibitors and adamantanes. *Antiviral Res* **73** 228-2231.

**Hurt AC, Holien JK, Barr IG. 2009.** In vitro generation of neuraminidase inhibitor resistance in A(H5N1) influenza viruses. *Antimicrob Agents Chemother* **53** 4433-40.

**Hurt AC, Lowther S, Middleton D, Barr IG. 2010.** Assessing the development of oseltamivir and zanamivir resistance in A(H5N1) influenza viruses using a ferret model. *Antiviral Res* **87** 361-366.

**Imai M, Watanabe T, Hatta M, Das SC, Ozawa M, Shinya K, Zhong G, Hanson A, Katsura H, Watanabe S, Li C, Kawakami E, Yamada S, Kiso M, Suzuki Y, Maher EA,**

**Neumann G, Kawaoka Y. 2012.** Experimental adaptation of an influenza H5 HA confers respiratory droplet transmission to a reassortant H5 HA/H1N1 virus in ferrets.

*Nature.* **486** 420-428.

**Ilyushina NA, Govorkova EA, Webster RG. 2005.** Detection of amantadine-resistant variants among avian influenza viruses isolated in North America and Asia. *Virology* **341** 102-106.

**Ilyushina NA, Seiler JP, Rehg JE, Webster RG, Govorkova EA. 2010.** Effect of neuraminidase inhibitor-resistant mutations on pathogenicity of clade 2.2 A/Turkey/15/06 (H5N1) influenza virus in ferrets. *PLoS Pathog* **6** e1000933.

**Ilyushina NA, Bovin NV, Webster RG. 2012.** Decreased neuraminidase activity is important for the adaptation of H5N1 influenza virus to human airway epithelium. *J Virol* **86** 4724-4733.

**Jackson D, Hossain MJ, Hickman D, Perez DR, Lamb RA. 2008.** A new influenza virus virulence determinant: the NS1 protein four C-terminal residues modulate pathogenicity. *Proc Natl Acad Sci U S A* **105** 4381-4386.

**Jiao P, Tian G, Li Y, Deng G, Jiang Y, Liu C, Liu W, Bu Z, Kawaoka Y, Chen H. 2008.** A single-amino-acid substitution in the NS1 protein changes the pathogenicity of H5N1 avian influenza viruses in mice. *J Virol* **82** 1146-1154.

**Kiso M, Ozawa M, Le MT, Imai H, Takahashi K, Kakugawa S, Noda T, Horimoto T, Kawaoka Y. 2011.** Effect of an asparagine-to-serine mutation at position 294 in neuraminidase on the pathogenicity of highly pathogenic H5N1 influenza A virus. *J Virol* **85** 4667-4672.

**Kuo RL, Krug RM. 2009.** Influenza a virus polymerase is an integral component of the CPSF30-NS1A protein complex in infected cells. *J Virol* **83** 1611-1616.

**Le MT, Wertheim HF, Nguyen HD, Taylor W, Hoang PV, Vuong CD, Nguyen HL, Nguyen HH, Nguyen TQ, Nguyen TV, Van TD, Ngoc BT, Bui TN, Nguyen BG, Nguyen LT, Luong ST, Phan PH, Pham HV, Nguyen T, Fox A, Nguyen CV, Do HQ, Crusat M, Farrar J, Nguyen HT, de Jong MD, Horby P. 2008.** Influenza A H5N1 clade 2.3.4 virus with a different antiviral susceptibility profile replaced clade 1 virus in humans in northern Vietnam. *PLoS One* **3** e3339.

**Le QM, Kiso M, Someya K, Sakai YT, Nguyen TH, Nguyen KH, Pham ND, Ngyen HH, Yamada S, Muramoto Y, Horimoto T, Takada A, Goto H, Suzuki T, Suzuki Y, Kawaoka Y. 2005.** Avian flu: isolation of drug-resistant H5N1 virus. *Nature* **437** 1108.

**Li Z, Chen H, Jiao P, Deng G, Tian G, Li Y, Hoffmann E, Webster RG, Matsuoka Y, Yu K. 2005.** Molecular basis of replication of duck H5N1 influenza viruses in a mammalian mouse model. *J Virol* **79** 12058-12064.

**Long JX, Peng DX, Liu YL, Wu YT, Liu XF. 2008.** Virulence of H5N1 avian influenza virus enhanced by a 15-nucleotide deletion in the viral nonstructural gene. *Virus Genes* **36** 471-478.

**Mase M, Tanimura N, Imada T, Okamatsu M, Tsukamoto K, Yamaguchi S. 2006.** Recent H5N1 avian influenza A virus increases rapidly in virulence to mice after a single passage in mice. *J Gen Virol* **87** 3655-3659.

**Matsuoka Y, Swayne DE, Thomas C, Rameix-Welti MA, Naffakh N, Warnes C, Altholtz M, Donis R, Subbarao K. 2009.** Neuraminidase stalk length and additional glycosylation of the hemagglutinin influence the virulence of influenza H5N1 viruses for mice. *J Virol* **83** 4704-4708.

**Naughtin M, Dyason JC, Mardy S, Sorn S, von Itzstein M, Buchy P. 2011.** Neuraminidase inhibitor sensitivity and receptor-binding specificity of Cambodian clade 1 highly pathogenic H5N1 influenza virus. *Antimicrob Agents Chemother* **55** 2004-2010.

**Puthavathana P, Auewarakul P, Charoenying PC, Sangsiriwut K, Pooruk P, Boonnak K, Khanyok R, Thawachsupa P, Kijphati R, Sawanpanyalert P. 2005.** Molecular characterization of the complete genome of human influenza H5N1 virus isolates from Thailand. *J Gen Virol* **86** 423-433.

**Seo SH, Hoffmann E, Webster RG. 2002.** Lethal H5N1 influenza viruses escape host anti-viral cytokine responses. *Nat Med* **8** 950-954.

**Smith AM, McCullers JA. 2013.** Molecular signatures of virulence in the PB1-F2 proteins of H5N1 influenza viruses. *Virus Res* **178** 146-50.

**Spesock A, Malur M, Hossain MJ, Chen LM, Njaa BL, Davis CT, Lipatov AS, York IA, Krug RM, Donis RO. 2011.** The virulence of 1997 H5N1 influenza viruses in the mouse model is increased by correcting a defect in their NS1 proteins. *J Virol* **85** 7048-7058.

**Watanabe Y, Ibrahim MS, Ellakany HF, Kawashita N, Mizuike R, Hiramatsu H, Sriwilaijaroen N, Takagi T, Suzuki Y, Ikuta K. 2011.** Acquisition of human-type receptor binding specificity by new H5N1 influenza virus sublineages during their emergence in birds in Egypt. *PLoS Pathog* **7** e1002068.

**World Health Organization.** Summary table of neuraminidase amino acid substitutions associated with reduced inhibition by neuraminidase inhibitors (NAI). Last updated 21 October 2016. <http://www.who.int/influenza/gisrs_laboratory/antiviral_susceptibility/avwg2014_nai_substitution_table.pdf?ua=1> (Accessed 15 July 2017).

**Yamada S, Suzuki Y, Suzuki T, Le MQ, Nidom CA, Sakai-Tagawa Y, Muramoto Y, Ito M, Kiso M, Horimoto T, Shinya K, Sawada T, Kiso M, Usui T, Murata T, Lin Y, Hay A, Haire LF, Stevens DJ, Russell RJ, Gamblin SJ, Skehel JJ, Kawaoka Y. 2006.** Haemagglutinin mutations responsible for the binding of H5N1 influenza A viruses to human-type receptors. *Nature* **444** 378-382.

**Yamada S, Hatta M, Staker BL, Watanabe S, Imai M, Shinya K, Sakai-Tagawa Y, Ito M, Ozawa M, Watanabe T, Sakabe S, Li C, Kim JH, Myler PJ, Phan I, Raymond A, Smith E, Stacy R, Nidom CA, Lank SM, Wiseman RW, Bimber BN, O'Connor DH, Neumann G, Stewart LJ, Kawaoka Y. 2010.** Biological and structural characterization of a host-adapting amino acid in influenza virus. *PLoS Pathog* **6** e1001034.

**Yen HL, Ilyushina NA, Salomon R, Hoffmann E, Webster RG, Govorkova EA. 2007.** Neuraminidase inhibitor-resistant recombinant A/Vietnam/1203/04 (H5N1) influenza viruses retain their replication efficiency and pathogenicity in vitro and in vivo. *J Virol* **81** 12418-12426.

**Zhou H, Yu Z, Hu Y, Tu J, Zou W, Peng Y, Zhu J, Li Y, Zhang A, Yu Z, Ye Z, Chen H, Jin M. 2009.** The special neuraminidase stalk-motif responsible for increased virulence and pathogenesis of H5N1 influenza A virus. *PLoS One* **4** e6277.
